# A single amino acid substitution in Fibronectin Binding protein A (FnBPA) governs *Staphylococcus aureus* virulence via host transglutaminase-mediated fibrin crosslinking

**DOI:** 10.1371/journal.ppat.1013743

**Published:** 2025-12-01

**Authors:** Chiara Motta, Mary Turley, Giulia Barbieri, Agata Famà, Francesco Coppolino, Elisa Bellan Menegussi, Joan A. Geoghegan, Concetta Beninati, Angelica Pellegrini, Giampiero Pietrocola

**Affiliations:** 1 Department of Molecular Medicine, University of Pavia, Pavia, Italy; 2 Department of Microbes, Infections and Microbiomes, College of Medicine and Health, University of Birmingham, Birmingham, United Kingdom; 3 Institute of Microbiology and Infection, University of Birmingham, Birmingham, United Kingdom; 4 Department of Biology and Biotechnology, University of Pavia, Pavia, Italy; 5 Department of Human Pathology, University of Messina, Messina, Italy; 6 Research Department, Fondazione IRCSS Policlinico San Matteo, Pavia, Italy; University of Tubingen, GERMANY

## Abstract

*Staphylococcus aureus* is an opportunistic pathogen that can cause different types of infections, ranging from skin lesions to life threatening diseases. Its ability to evade host immunity and establish persistent infections relies on effective adhesion to host tissues, mainly through interactions between bacterial surface proteins and extracellular matrix (ECM) components such as fibrinogen (Fbg). Here we describe a new pathogenic mechanism where *S. aureus* uses host transglutaminases to covalently anchor itself to Fbg. Specifically, human Factor XIII (FXIII) activated by *S. aureus* coagulase von Willebrand factor-binding protein (vWbp) crosslinks the bacterial adhesin Fibronectin Binding Protein A (FnBPA) to fibrin(ogen). Beyond vWbp-activated FXIII, also tissue transglutaminase 2 (TG2) is involved in a similar pathway. Previous biochemical studies showed that Gln103 in the N1 subdomain of FnBPA is the main reactive glutamine residue for FXIII-mediated crosslinking. We demonstrated that FnBPA protein mutants lacking Gln103, substituted with an alanine (Q103A) retained non-covalent Fbg binding via the classical Dock, Lock, and Latch mechanism, but failed to form covalent complexes. Furthermore, a mutant bacterial strain expressing FnBPA Q103A showed impaired incorporation into fibrin matrices. Also, *in vivo* experiments demonstrated that in a murine infection model the same Q103A mutant was less virulent, formed smaller dermonecrotic lesions and had lower bacterial loads than the wild-type strain. Importantly, also TG2, expressed in various tissues and upregulated during inflammation, crosslinks FnBPA to Fbg only in presence of Gln103, highlighting the broader physiological relevance of this mechanism beyond coagulation sites. Together, these results show a new virulence strategy where *S. aureus* uses vWbp to hijack host transglutaminase activity, stabilizing bacterial-host protein complexes through covalent FnBPA-Fbg interactions.

## Author summary

*Staphylococcus aureus* is an opportunistic pathogen capable of causing several infections. Its ability to colonize host tissues depends on adhesion to host proteins such as fibrinogen. In this study, we describe a novel mechanism by which *S. aureus* stabilizes its attachment: the bacterium exploits host transglutaminases, enzymes involved in clotting and tissue repair, to form covalent bonds between its surface protein FnBPA and fibrinogen. This process is mediated by the bacterial coagulase von Willebrand factor binding protein, that activates the host Factor XIII, promoting crosslinking. A key residue in FnBPA was previously identified as essential for crosslink formation. Here, we show that mutant proteins lacking this site retained non-covalent binding but failed to form covalent complexes. A bacterial strain expressing the same mutant protein exhibited reduced incorporation into fibrin matrices. Importantly, these mutants caused smaller lesions and lower bacterial loads than wild-type bacteria in a mouse infection model, highlighting the importance of covalent anchoring to the host for virulence. Interestingly, this mechanism involves not only clotting-associated Factor XIII but also the Tissue Transglutaminase 2, suggesting a broader relevance. These findings reveal a novel strategy and identify FnBPA-mediated covalent crosslinking as potential target for therapeutic intervention against antibiotic-resistant *S. aureus* infections.

## Introduction

*Staphylococcus aureus* is a major human pathogen, renowned for its versatility in causing a broad array of infections [1]. It is particularly concerning in both community and healthcare settings due to its ability to thrive in diverse environments. This pathogen is not only increasingly resistant to antibiotics but is also a master of immune evasion, circumventing nearly every aspect of the host’s defense mechanisms [2].

Moreover, *S. aureus* stands as the leading cause of infective endocarditis, a serious heart infection where it adheres to fibrin-platelet clots on damaged heart valves, forming vegetations that complicate treatment [3,4]. A hallmark of *S. aureus* is its ability to colonize human tissues following vascular injury, attributed to microbial surface components recognizing adhesive matrix molecules (MSCRAMMs) [5]. These receptors facilitate the binding of *S. aureus* to human fibrin(ogen) [6–8], fibronectin [9,10], and collagen [11], representing a critical step in the initiation of infection.

**Fig 1:**
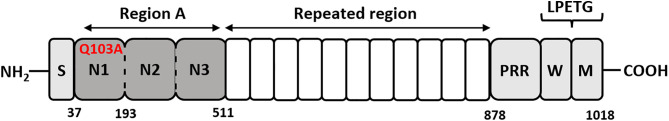
Structural organization of FnBPA. Structurally, FnBPA begins with an N-terminal signal sequence (S), which is followed by Region A (37-511), responsible for fibrinogen binding. Region A is composed of three subdomains (N1, N2, and N3). At position 103 inside the N1 subdomain there is a key reactive glutamine, which mediates transglutaminase reactions. As part of our study, this specific residue was replaced with an alanine via site-directed mutagenesis, resulting in the Q103A variant. The C-terminal region, named also Repeated region (511-878) is composed of multiple fibronectin-binding motifs arranged in tandem. Toward the end of the C-terminus there are proline-rich repeats (PRR), followed by wall-associated (W) and membrane-spanning (M) domains. The LPETG motif is recognized by the sortase enzyme for cell wall anchoring.

Among the MSCRAMMs, fibronectin-binding protein A (FnBPA) and its homolog FnBPB, play a central role in host infection [12]. FnBPA is composed of two major domains, the N-terminal and C-terminal. The N-terminal domain, also called Region A, is further divided into three subdomains (N1, N2, and N3) that form IgG-like folds [13], and through the “dock, lock, and latch” (DLL) mechanism it is able to interact with ligands such as fibrinogen (Fbg) [8]. Meanwhile, the C-terminal domain is composed of fibronectin-binding repeats (FnBRs), that engage the N-terminal type I modules of fibronectin in a ‘tandem β-zipper’ fashion to support bacterial invasion [14] (Fig 1).

Previous studies have demonstrated that FnBPA is specifically recognized by and serves as a substrate for coagulation factor XIIIa. The activation of factor XIII by thrombin in the presence of calcium ions leads to the formation of cross-links between Fbg and FnBPA [15]. Additionally, *S. aureus* secrets hemostatic-modulating virulence factors [16] such as coagulase (Coa) [17] and von Willebrand factor-binding protein (vWbp) [18–20], both of which can non-proteolytically activate prothrombin (ProT) to generate thrombin. The resulting thrombin cleaves Fbg into fibrin, promoting the formation of fibrin cables [21–23].

This mechanism plays a significant role in enhancing bacterial pathogenicity, as demonstrated in various animal infection models [22–24]. Coa and vWbp share structural and functional similarities, particularly in their N-terminal regions that activate ProT [25]. Both proteins bind Fbg through their N- and C-terminal domains, but with varying affinities [26]. Notably, vWbp also interacts with FXIII via its N-terminal region [23] and once secreted, it binds electrostatically to the bacterial surface [27]. This interaction creates a focal point for coagulation factor recruitment, centered around the expressed FnBPA, and promotes the localized formation of fibrin networks that contribute to bacterial adhesion and immune evasion.

In our previous study, we discovered that vWbp-activated FXIII catalyses the formation of high molecular heteropolymers between FnBPA and Fbg. Additionally, similar outcomes were observed when another transglutaminase, named tissue transglutaminase-2 (TG2), was evaluated for its ability to cross-link FnBPA and Fbg [27]. This enzyme can be found in fibroblasts, vascular endothelium, smooth muscle cells, and in the extracellular matrix (ECM) of various tissues and of the arterial walls [28].

In our prior work, cross-linking sites were mapped to the α-chain of Fbg and the N1 subdomain of FnBPA, respectively [27]. These findings are consistent with the work of Severina *et al*., in which they identified the FnBPA residue glutamine103, located in the N1 subdomain, as a major cross-linking site for thrombin-activated FXIII [29].

Along this line, we decided to produce mutant recombinant FnBPA proteins, by substituting the reactive glutamine (position 103 of the mature form of the protein) with an alanine (Q103A) (Fig 1) to investigate the role of this residue in the cross-linking to Fbg through the action of vWbp-activated FXIII or TG2.

To conclude, we also produced through site-directed mutagenesis a Q103A mutant of *S. aureus* to analyse the covalent incorporation of the whole bacterial cell to Fbg.

## Results

### Conservation of residue Q103 in FnBPA cross-linking with Fbg across *S. aureus* clonal complexes

**Fig 2:**
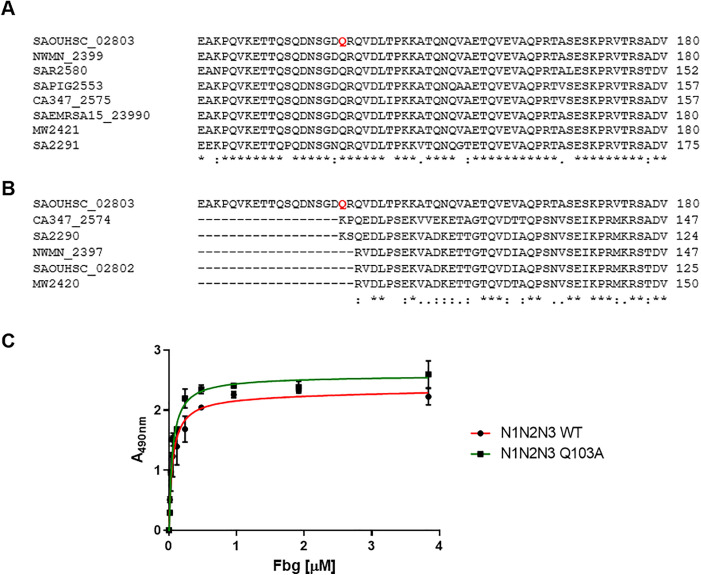
Conservation and functional relevance of the Q103 residue in FnBPA. (A) Multiple sequence alignment of *fnbA* gene sequences from representative *S. aureus* strains across major clonal complexes to determine the conservation of the Q103 residue (highlighted), implicated in bacterially-mediated cross-linking with fibrinogen (Fbg). (B) Multiple sequence alignment of *fnbB* sequences from corresponding clonal complexes with *fnbA* (subsp.8325). Alignments in Fig 2A and 2B were carried out with ClustalOmega. Gene sequences were retrieved from KEGG GENOME database with the following entries: T00557 (subsp. Newman - CC8, NWMN_2397/ NWMN_2397); T00324 (subsp. 8325 - CC8, SAOUHSC_02803/ SAOUHSC_02802); T00086 (subsp. MW2 – CC1, MW2041/MW2040); T02702 (subsp. CA347 – CC45, CA347_2575/ CA347_2574); T00182 (subsp. MRSA252 – CC30, SAR2580); T00051 (subsp. N315 – CC5, SA2291/SA2290); T02059 (subsp. HO 5096 0412 - CC22, SAEMRSA15_23990); T02071 (subsp. ST398 – CC398, SAPIG2553) (C) Saturation binding curves of recombinant WT and Q103A-mutated FnBPA N1N2N3 to Fbg. Microtiter plates were coated with each protein variant and incubated with increasing concentrations of Fbg. Detection was performed using anti-Fbg antibody and HRP-conjugated secondary antibody. Data are expressed as means ± S.D. of tests performed in triplicate.

Severina *et al*. demonstrated that thrombin-activated FXIII catalyses covalent cross-linking between full-length FnBPA and Fbg and proposed the Q103 residue as a major cross-linking site [29]. To begin, we investigated whether this residue is conserved in the FnBPA sequence expressed by different *S. aureus* clonal complexes. To this end, we performed a multiple sequence alignment of the amino acid sequences of the *fnbA* gene and found that, across all strains analyzed, Q103 is highly conserved in the major clonal lineages (Fig 2A). Next, to further evaluate the functional relevance of the Q103 residue, we extended our analysis by aligning the amino acid sequences of all seven known isoforms of the FnBPA protein expressed by *S. aureus*. These isoforms, which differ primarily in the number and arrangement of fibronectin-binding repeats, have been previously characterized and shown to be expressed across various *S. aureus* strains [30]. The multiple sequence alignment revealed that Q103 is strictly conserved across all isoforms, despite overall sequence variability among them (S1 Fig).

To assess the importance of the Q103 residue, we aligned the sequences of *fnbB* from several clonal complexes and verified that this residue, present in the *fnbA* gene of the reference strain (*S. aureus* 8325–4, CC8) is absent in *fnbB* (Fig 2B). This result is consistent with our previously published data [27], which showed that FnBPB does not cross-link with fibrin(ogen), in contrast to FnBPA, thereby confirming the critical role of Q103 in cross-link formation.

Given the high degree of Q103 conservation, we proceeded to produce recombinant mutant domains of FnBPA, where Q103 was replaced by an alanine residue (Q103A). Specifically, we produced the N1 subdomain, where Q103 is located, as well as the N1N2N3 domain of FnBPA, (Fig 2C). As reported in the literature, the A region of FnBPA is capable of binding to Fbg through the N2N3 subdomains, whereas the N1 subdomain does not [8]. To verify that the Q103A substitution does not alter the binding of the N1N2N3 domain to Fbg, we constructed saturation binding curves by comparing the binding of the mutated protein with that of the WT. For this purpose, microtiter wells were coated with either the wild-type (WT) or Q103A form of the protein, incubated with increasing concentrations of soluble Fbg, and then the complex was revealed with an anti-Fbg antibody. As shown in Fig 2C, the Q103A substitution does not affect the binding of the N1N2N3 region to Fbg.

### The role of Q103 in the covalent binding of FnBPA to fibrin(ogen): Effects of the Q103A mutation on FXIIIa-mediated crosslinking

**Fig 3:**
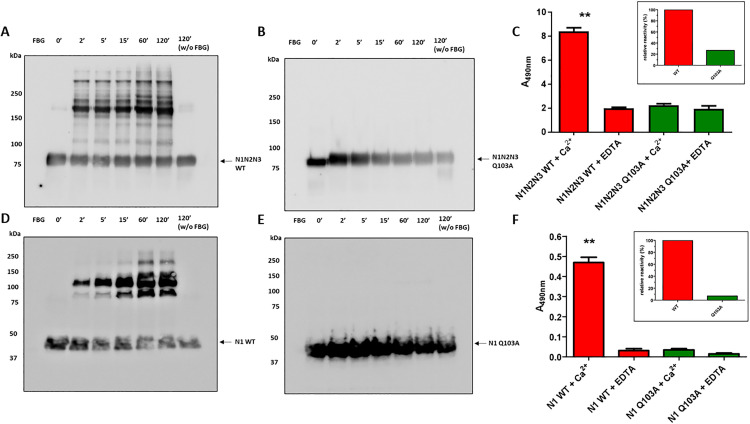
Role of Q103A residue in vWbp-mediated cross-linking of FnBPA to fibrin(ogen). (A–B) Cross-link formation of recombinant N1N2N3 domain of FnBPA WT (A) or Q103A mutant (B) to fibrin(ogen) by vWbp-activated FXIII was analyzed by Western Blotting. Reaction mixtures were incubated for increasing time points (0–120 min) in the presence of calcium, separated by SDS-PAGE under reducing conditions and then transferred to PVDF membranes, then probed with anti-FnBPA IgG and HRP-conjugated secondary antibody. Lane 8 in both panels lacks fibrin(ogen); lane 1 contains only fibrin(ogen) Arrows show the positions of FnBPA WT (A) or FnBPA Q103A. Molecular mass markers (kDa) are indicated on the left. Data is representative of three independent experiments. (C) ELISA analysis of fibrin(ogen) crosslinking with WT and Q103A N1N2N3 domains in the presence of Ca²⁺ or EDTA. Microtiters wells were coated with fibrinogen and then probed with mixtures containing FnBPA WT or Q103A, vWbp, ProT, FXIII and either calcium chloride or EDTA. Binding was measured using anti-FnBPA IgG and HRP-conjugated secondary antibody. Statistically significant difference is reported (**P < 0.01). Inset: binding of the mutant protein was reduced to approximately 30% of the reactivity observed for the FnBPA WT. Data are expressed as means ± S.D. of triplicate tests. (D–E) Cross-link formation of recombinant N1 subdomain of FnBPA WT (D) or Q103A (E) to fibrin(ogen) by vWbp-activated FXIII, analyzed as described for panels A–B. Arrows show the positions of FnBPA WT or FnBPA Q103A. (F) ELISA analysis of fibrin(ogen) crosslinking with WT and Q103A N1 domains in the presence of Ca²⁺ or EDTA. Statistical analysis is reported (**P < 0.01). Inset: WT signal in presence of Ca² ⁺ ions is > 90% higher than mutant.

In our previous work, we demonstrated that the N1N2N3 region of FnBPA, particularly the N1 subdomain, is capable of covalently binding to fibrin(ogen) through the action of vWbp-activated FXIII, resulting in the formation of high molecular weight heterocomplexes [27]. To assess whether the mutated recombinant FnBPA still retains the ability to be crosslinked to fibrin(ogen) by bacterially-activated FXIII, both WT and Q103A forms of the N1N2N3 protein were incubated with vWbp-activated FXIII and fibrin(ogen) for increasing periods of time (0–120 minutes) in presence of calcium ions. The reaction products were then subjected to SDS-PAGE under reducing conditions and transferred to a PVDF membrane by Western blotting. The membrane was subsequently incubated with an anti-FnBPA IgG to detect the formation of heterocomplexes with fibrin(ogen). The results demonstrated that the WT protein successfully covalently binds to fibrin(ogen) via FXIII activation by vWbp, generating high molecular weight complexes, thus confirming our previous findings (Fig 3A). In contrast, when the FnBPA Q103A mutant was incubated in the same reaction, the formation of high molecular weight complexes was completely inhibited (Fig 3B). In mixtures where Fbg was omitted, no high molecular weight complexes were detected, confirming the validity of the assay (lane 8, Fig 3A,3B). The specificity of the anti-FnBPA antibody was verified by loading a mixture that excluded all components except Fbg (lane 1, Fig 3A,3B).

To compare the interaction levels of N1N2N3 WT and Q103A mutant proteins with fibrin(ogen) in the presence or absence of activated FXIII, ELISA-based assays were conducted. Mixtures containing vWbp-activated FXIII, vWbp-generated thrombin, and either WT or mutated FnBPA were incubated on fibrin(ogen)-coated microtiter wells in the presence of calcium or EDTA. Calcium was added as a necessary cofactor for FXIII activation, while EDTA, by sequestering calcium, inactivated FXIII. Consequently, protein binding to fibrin(ogen) in the presence of calcium reflects the combined contribution of noncovalent interactions, mediated by the DLL mechanism, and covalent cross-links catalyzed by FXIII. Conversely, in the presence of EDTA, only noncovalent interactions between FnBPA and fibrin(ogen) are observed. The results show that the binding level of N1N2N3 WT to fibrin(ogen) in the presence of calcium was significantly higher (**P < 0.01), approximately 4-fold, compared to the binding observed in the presence of EDTA (Fig 3C). This indicates that FXIII-mediated cross-link formation substantially enhances the incorporation of N1N2N3 WT into fibrin(ogen). In contrast, the Q103A mutant showed no significant difference in binding to fibrin(ogen) in presence or absence of calcium, confirming that the Q103A mutation prevents FXIII-mediated cross-link formation (Fig 3C). This result suggests that the binding of the mutant protein is exclusively due to non-covalent interactions (Fig 3C).

Ultimately, in presence of calcium, the mutant protein showed a significant decrease of binding, which accounted for only 30% of the reactivity displayed by the FnBPA WT (Fig 3C, inset). To confirm these results, we performed additional assays to compare the ability of the recombinant N1 WT subdomain, and the mutated N1 Q103A subdomain to form cross-links with fibrin(ogen) through the action of FXIII activated by vWbp. As shown in Fig 3D, the N1 WT subdomain retains its ability to form high-molecular-weight heterocomplexes with fibrin(ogen) through FXIII activation by vWbp. In contrast, the Q103A mutation completely abolishes the formation of these complexes (Fig 3E).

The ELISA assay also confirmed that the substitution of Q103 significantly disrupts the interaction between the N1 subdomain and fibrin(ogen) via FXIII activation by vWbp. Since the N1 subdomain does not interact with Fbg through non-covalent interactions, as the N1N2N3 domain does, the results in Fig 3F clearly show that the N1 subdomain is able to interact with fibrin(ogen) only in the presence of FXIII activated by vWbp and Ca^2+^, via cross-linking. In fact, no interaction of the WT protein was observed in the presence of EDTA, nor was there any interaction of the Q103A protein in the presence or absence of calcium. Similarly to what was previously observed, the interaction of N1 WT to fibrin(ogen) in the presence of calcium was significantly higher (**P < 0.01), approximately 15-fold, compared to what observed in the presence of EDTA (Fig 3F). Instead, mixtures containing calcium ions or EDTA with N1 Q103A showed almost identical absorbance values, indicating that the Q103A mutation is sufficient to inhibit the formation of covalent complexes (Fig 3F). In particular, when the mixtures were incubated with calcium, the binding of N1 WT to fibrin(ogen) was more than 90% higher than that of the mutant protein, further demonstrating the key role of Q103 (Fig 3F, inset).

### Effects of the Q103A mutation on TG2-mediated cross-linking

**Fig 4:**
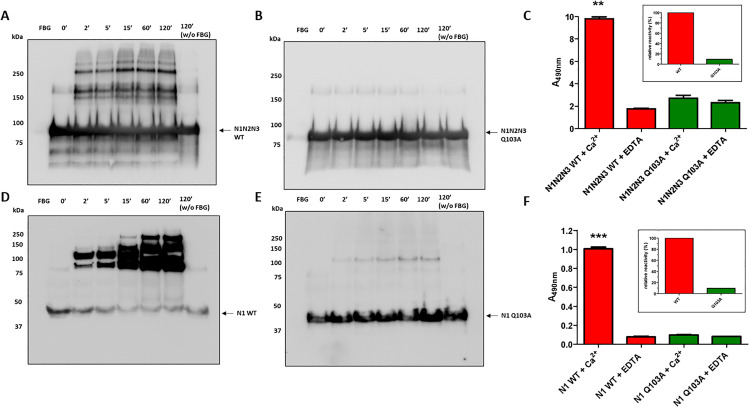
Role of Q103A residue in TG2- mediated cross-linking of FnBPA to fibrinogen. (A–B) Cross-link formation of recombinant N1N2N3 domain of FnBPA WT (A) or Q103A mutant (B) to fibrinogen by TG2 was analyzed by Western Blotting. Arrows show the position of the N1 WT (A) or Q103A (B) subdomain of FnBPA. Reaction mixtures were incubated for increasing time intervals (0–120 min) in the presence of calcium, separated by SDS-PAGE and transferred to PVDF membranes, then probed with anti-FnBPA IgG and HRP-conjugated secondary antibody. Lane 8 in both panels lacks fibrinogen; lane 1 contains only fibrinogen. Molecular mass markers (kDa) are indicated on the left. Data is representative of three independent experiments. (C) ELISA analysis of fibrin(ogen) crosslinking with WT and Q103A N1N2N3 domains in the presence of Ca²⁺ or EDTA by TG2. Microtiters wells were coated with fibrinogen and then incubated with mixtures containing FnBPA WT or Q103A, vWbp, ProT, FXIII and either calcium chloride or EDTA. Binding was measured using anti-FnBPA IgG and HRP-conjugated secondary antibody. Statistically significant difference is reported (**P < 0.01). Inset: binding of the mutant protein was reduced to about 30% of the interaction of WT FnBPA. Data are expressed as means ± **S.**D. of triplicate tests.(D–E) Cross-link formation of recombinant N1 subdomain of FnBPA WT (D) or Q103A (E) to fibrinogen by TG2, analyzed as described for panels A–B.(F) ELISA analysis of fibrin(ogen) crosslinking with WT and Q103A N1 domains by the action of TG2 in the presence of Ca²⁺ or EDTA. Statistically significant differences are reported (***P < 0.001). Inset: binding of WT protein was about 90% greater of the FnBPA Q103A.

As recently published in our previous work, the most ubiquitously expressed member of the tissue transglutaminase family, TG2, is able to catalyse the formation of high molecular weight complexes between FnBPA N1 and Fbg [27]. To determine whether the mutated recombinant FnBPA retains its ability to be crosslinked to Fbg by TG2, WT and Q103A mutant N1N2N3 proteins were incubated with TG2 in presence of calcium ions for up to 120 minutes. Reaction products were subjected to SDS-PAGE under reducing conditions, transferred to a PVDF membrane, and analyzed by Western blotting with an anti-FnBPA antibody to detect heterocomplex formation with Fbg. The results showed that the WT protein was able to generate high molecular weight complexes with Fbg through TG2-mediated cross-linking (Fig 4A), consistent with previous findings. In contrast, the Q103A mutant failed to form these complexes under the same conditions (Fig 4B).

To evaluate the interaction levels of N1N2N3 WT and Q103A mutant proteins with Fbg in the presence or absence of TG2, ELISA-based assays were performed as above. Mixtures containing TG2, and either WT or mutant FnBPA were incubated on Fbg-coated microtiter plates in the presence of calcium or EDTA. The findings revealed that N1N2N3 WT exhibited significantly higher binding to Fbg in the presence of calcium (approximately five times greater, **P < 0.01) compared to binding observed in the presence of EDTA (Fig 4C). This indicates that TG2-mediated cross-link formation plays a major role in enhancing the incorporation of N1N2N3 WT into Fbg. On the other hand, the Q103A mutant showed no significant difference in binding between conditions with or without calcium, demonstrating that the mutation prevents TG2-mediated cross-linking (Fig 4C). Finally, when calcium was present, the Q103A mutant displayed a marked reduction in binding, accounting for only 30% of the binding observed for the FnBPA WT (Fig 4C, inset). This suggests that while the mutant retains some binding affinity, the lack of TG2-mediated cross-linking severely limits its interaction with Fbg.

To validate our previous findings, we decided to perform the same experiments with the N1 domain of FnBPA. We incubated N1 WT or N1 Q103A with Fbg in the presence of TG2 and calcium. The resulting samples were analyzed via SDS-PAGE, followed by Western blotting using an anti-FnBPA antibody. As expected, the WT protein formed high molecular weight complexes, consistent with our prior results (Fig 4D). In contrast, the Q103A mutant completely lacked the ability to form such complexes under identical conditions (Fig 4E).

To assess the levels of binding of the WT and mutated protein, we employed the ELISA-based assay described earlier. We incubated N1 WT or Q103A with TG2 on Fbg-coated microtiter wells in the presence of either calcium chloride or EDTA. Consistent with previous observations, the N1 WT protein demonstrated significantly higher binding to Fbg in the presence of calcium (***P < 0.001, approximately 13-fold) compared to EDTA (Fig 4F). In contrast, mixtures containing the Q103A mutant showed comparable absorbance values regardless the presence of calcium or EDTA, indicating that the Q103A mutation effectively prevents covalent complex formation (Fig 4F). Notably, in calcium-containing mixtures, the binding of N1 WT to fibrin(ogen) was over 90% greater than that of the Q103A mutant, underscoring the critical role of Q103 in this interaction (Fig 4E, inset).

### Interaction with Fbg of mutant *Staphylococcus aureus* SH10004X (pFnBA4_Q103A) is unaltered, but cannot be covalently incorporated into fibrin by vWbp-activated FXIII

**Fig 5:**
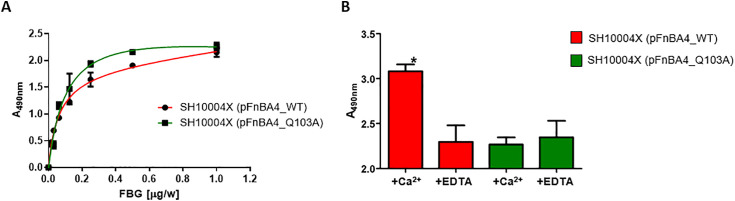
Impact of the Q103A substitution in FnBPA on *S. aureus* SH10004X binding to fibrinogen and cross-linking to fibrin. (A) Binding of fibrinogen (Fbg) to *S. aureus* SH10004X strains expressing either WT or Q103A-mutated FnBPA. Microtiter wells were coated with diluted bacterial suspensions (OD = 1) and incubated with serial dilutions of Fbg. Binding was detected using an anti-Fbg antibody and an HRP-conjugated secondary antibody. Data are expressed as means ± S.D. of tests performed in triplicate. (B) FXIII-mediated incorporation of *S. aureus* SH10004X (pFnBA4_WT) or (pFnBA4_Q103A) fibrin in presence of calcium or EDTA. Microtiter wells were coated with Fbg and incubated with mixtures containing *S. aureus* SH10004X strains expressing either WT or Q103A-mutated FnBPA, vWbp, FXIII, ProT and either calcium ions or EDTA. Binding of bacteria to fibrin was then detected using an anti-*S. aureus* antibody and an HRP-conjugated secondary antibody. Statistically significant differences are reported (*P < 0.05). Data are expressed as means ± **S.**D. of triplicate tests.

In view of the results obtained with the recombinant mutant proteins, we decided to continue our investigation by expressing FnBPA Q103A on the surface of *S. aureus*. Strain SH1000 deficient in *clfA*, *clfB, fnbA* and *fnbB* (SH10004X) cannot bind to Fbg and was used as a host for plasmid expression. Plasmid pFnBA4 containing the whole FnBPA protein [31] was subjected to site-directed mutagenesis to obtain the substitution. Subsequently, *S. aureus* SH10004X was transformed with the mutated construct, following an adapted protocol from Lofblom *et al* [32]. Microtiter wells were coated with the *S. aureus* SH10004X (pFnBPA4_WT) or SH10004X (pFnBPA4_Q103A) and incubated with serial dilutions of Fbg. As shown in Fig 5A, SH10004X (pFnBA4_Q103A) maintained the same capture of Fbg as the strain carrying the wild-type protein.

Since we demonstrated the ability of the recombinant mutant proteins to inhibit the crosslinking to fibrin(ogen), we proceeded with testing the covalent incorporation of the SH10004X (pFnBPA4_Q103A) into fibrin by an ELISA assay. Microtiter wells coated with Fbg were incubated with mixtures containing vWbp-activated FXIII and the strain carrying the wild type or the mutated FnBPA, supplemented with calcium ions or EDTA, and detected interacting bacteria with anti-*S. aureus* IgG. Similarly to the previous experiments with the recombinant proteins, if SH10004X (pFnBPA4_WT) was incubated in presence of calcium, the absorbance values were higher compared with those with EDTA, reflecting the covalent incorporation of the bacterial cell in the fibrin mesh. Instead, the mutant Q103A strain showed the same absorbance levels regardless of the presence of calcium or EDTA (Fig 5B). This result demonstrates that this single amino acid substitution can inhibit the cross-linking of *S. aureus* into fibrin.

### The Q103A substitution significantly impairs the ability of FnBPA to promote evasion of host defenses and the induction of tissue damage by *S. aureus*

**Fig 6:**
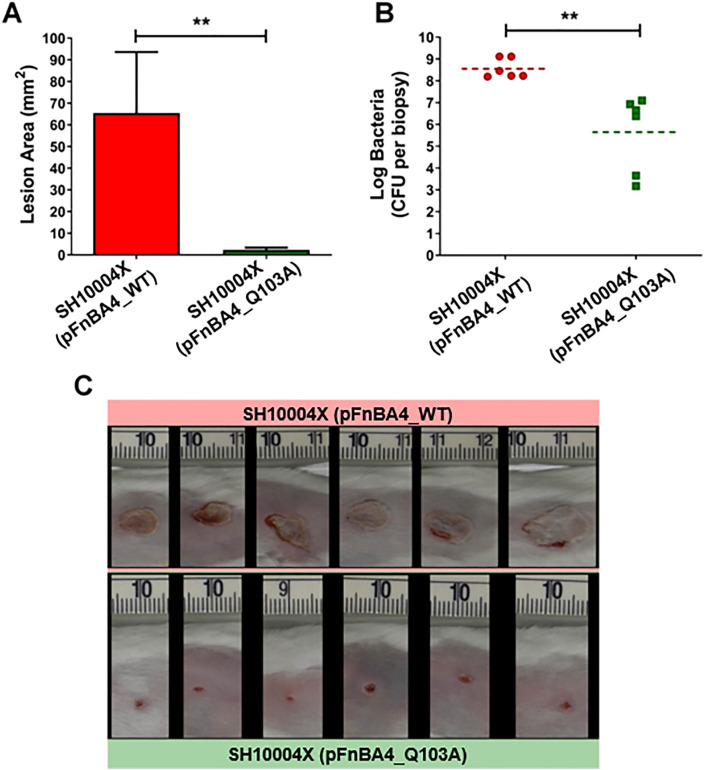
*In vivo* impact of Q103A mutation in a murine dermonecrosis model. (A) Mean lesions size (mm²) were measured in mice infected with *S. aureus* SH10004X strains expressing either WT or Q103A-mutated FnBPA at day 7 post-infection. (B) Bacterial load per biopsy was quantified by CFU counts at day 7 post-infection. Each dot represents an individual mouse. Bars indicate the means. Statistically significant differences were calculated (**p < 0.01). (C) Photographs of lesions at day 7 post-infection in mice infected with *S. aureus* SH10004X strains expressing either WT or Q103A-mutated FnBPA.

To assess the mutant’s virulence, we utilized a well-established mouse model of necrotic dermatitis, which mimics several aspects of human staphylococcal skin infections [33]. In this model, bacteria are injected subcutaneously, and the progression of necrotic ulcers is monitored over time, along with bacterial load assessments in skin biopsies. As shown in Fig 6, the lesion area, a reliable marker of infection severity, was significantly smaller in mice infected with SH10004X (pFnBPA4_Q103A) compared to those infected with the wild-type strain. Additionally, bacterial burden was found to be more than 10⁵-fold higher in skin biopsies from mice infected with the wild-type strain. *S. aureus* alpha toxin, or α-hemolysin (Hla) is a pore-forming toxin which causes dermonecrosis in mouse models, representing a critical factor for pathogenesis of skin and soft tissue infections [34]. In order to exclude that the different dermonecrosis levels observed in mice could be due to an altered production of bacterial alpha-toxin, we conducted western blot analyses on supernatants from bacterial cultures to verify that both the SH10004X (pFnBPA4_WT) and SH10004X (pFnBPA4_Q103A) bacterial strains produce the same amounts of alpha-toxin. Densitometric analysis of immunoblot signals allowed us to quantify at a protein level the expression of the Hla alpha-toxin secreted from the bacterial strains. The results obtained revealed that both bacterial strains release the same amount of alpha-toxin (S2 Fig).

Taken together, these results demonstrate that the Q103A mutation substantially diminishes the virulence of *S. aureu*s.

## Discussion

The ability of *S. aureus* to adhere to host Fbg and incorporate into the fibrin network is a key determinant of its pathogenicity. The Fbg binding proteins ClfA, ClfB, FnBPA and FnBPB play complementary roles during different stages of infection, being involved in initial stages of tissue colonization, and in more deep-rooted infections, such as during biofilm formation [35–37]. Among them, FnBPA plays a central role in mediating bacterial attachment to Fbg and contributing to the formation of fibrin-based matrices that stabilizes colonization, especially in damaged tissue or medical implants [38,39]. Recent evidence has shown that FnBPA not only binds to Fbg via non-covalent interactions but can also be covalently cross-linked to fibrin(ogen) through the action of host transglutaminase such as FXIII and TG2 [27,29]. The potential contribution of the other fibrinogen-binding proteins in cross-link formation was addressed in our previous study and demonstrated that they are not involved in the crosslinking mediated by vWbp-activated FXIII [27].

In the present study, we sought to decipher the molecular mechanism underlying FnBPA covalent interaction with fibrin(ogen), focusing on the glutamine residue Q103, previously proposed as a critical FXIIIa target site [27,29].

Sequence analysis confirmed that Q103 is highly conserved across major *S. aureus* clonal complexes and the seven isoforms of FnBPA described by Loughman *et al*. (2008), suggesting evolutionary pressure to maintain this residue [30]. In contrast, FnBPB, which does not cross-link to Fbg [27] lacks a corresponding glutamine residue, strengthening the hypothesis that Q103 is functionally indispensable for covalent cross-linking to Fbg.

To directly test this, we generated recombinant FnBPA variants bearing a Q103A substitution (Fig 1). This mutation did not impair non-covalent binding to Fbg, in agreement with the known Dock, Lock, and Latch (DLL) mechanism that involves the N2N3 subdomains of region A [8]. As Q103 is located within the N1 subdomain (Fig 1) it is unsurprising that its substitution does not disrupt the initial adhesive interaction. However, the Q103A mutation abolished covalent crosslinking to Fbg when mediated by both vWbp-activated FXIII and TG2. These findings were consistently observed using both the full region A (N1N2N3) and the isolated N1 subdomain of FnBPA, as assessed by Western blot and ELISA.

Importantly, this mechanism is not unique to *S. aureus*. Other Gram-positive pathogens, including Group B *Streptococcus* and *Streptococcus pyogenes,* use similar strategies to exploit host transglutaminases for protein cross-linking to host matrices [40,41]. In contrast, Gram-negative pathogens such as *Escherichia coli* form fibrin-like deposits on their surface via non-covalent mechanisms that do not rely on host enzymatic activity [42].

These results highlight the functional importance of Q103 and its evolutionary conservation. Glutamine-rich regions (compositional biased regions, or CBRs) are notably conserved in pathogenic bacteria and are believed to play a role in virulence and host-pathogen interactions [43].

Our results show that the Q103A mutation significantly inhibits the formation of covalent crosslinks between FnBPA and Fbg in the presence of both vWbp-activated FXIII and TG2. This supports the hypothesis that Q103 serves as a glutaminyl donor for transglutaminase activity or contributes to maintaining a conformation competent for crosslinking. Remarkably, this mutation does not affect non-covalent Fbg binding, reaffirming that Q103 is dispensable for initial adhesion but essential for covalent incorporation.

As already mentioned, the Q103A mutation also inhibits crosslinking when TG2 is used instead of FXIII. TG2 is a multifunctional enzyme with established roles in protein crosslinking, and it has been shown to contribute to fibrinogen crosslinking in various contexts [28]. Its role in bacterial infections is increasingly recognized. For instance, TG2 promotes bacterial survival in *Chlamydia trachomatis* infection models, and its inhibition reduces bacterial replication [44]. TG2 is upregulated in macrophages during sepsis, where it supports inflammatory responses and macrophage survival via NF-κB signalling [45]. This broader context of innate immune activation is consistent with our previous findings, which highlighted the role of endosomal Toll-like receptors in shaping the host response to *S. aureus* infection [33].

The physiological relevance of Q103 is further demonstrated by the *S. aureus* SH1000 4X (pFnBPA4_Q103A) strain, which failed to be incorporated into fibrin in the presence of vWbp-activated FXIII. This aligns with our previous data, suggesting that TG2 and FXIII-mediated crosslinking promotes *S. aureus* virulence and persistence in tissue [27].

This hypothesis is strongly supported by our *in vivo* findings, showing that the Q103A mutant exhibits markedly reduced lesion formation and bacterial burden in a murine model of dermonecrosis. Loss of crosslinking capability impairs the integration of the bacterium into the fibrin matrix, potentially reducing its ability to resist immune clearance or colonize damaged tissues. Although our *in vitro* studies were performed with human fibrinogen and transglutaminase, while the *in vivo* experiments relied on murine components, it is well established that the coagulation systems of humans and mice share a high degree of structural and functional conservation. Furthermore, many coagulation factors are homologous in sequence and function across the two species [46,47]. Studies have demonstrated that murine coagulation factors, including ProT and FXIII, can interact with their human counterparts, further supporting the translational relevance of murine models [48,49].

However, the precise role of host transglutaminases in *S. aureus* infections remains to be fully elucidated. On one hand, TG2 and FXIII-mediated fibrin crosslinking may function as a host defense mechanism, restricting bacterial dissemination by entrapping pathogens in fibrin-rich clots. This is supported by studies showing enhanced susceptibility to infection in FXIII-deficient mice [40]. This suggests that transglutaminases are part of the host’s innate defense mechanisms. On the other hand, *S. aureus* may exploit this host defense mechanism to its advantage. The formation of FnBPA-Fbg covalent heteropolymers may enhance adhesion in Fbg-rich environments such as inflamed tissue or implanted biomaterials, contributing to persistent infection [50,51] In summary, our data demonstrate that Q103 can regulate the covalent incorporation of *S. aureus* into the host fibrin network. This process, mediated by host transglutaminases, represents a molecular interaction in which bacterial proteins exploit host enzymes to promote stable colonization. The human transglutaminases play a dual role: acting both as barriers to bacterial spread and as facilitators of adhesion, highlighting the delicate balance in host-pathogen interactions.

Targeting this covalent interaction represents a promising therapeutic avenue, especially in the context of invasive infections and biofilm-associated disease. Future studies should explore the dynamics of transglutaminase activity in vivo, particularly within inflamed or immune-privileged tissues. Understanding how FnBPA crosslinking modulates infection outcomes may guide the development of anti-adhesive therapies that disarm *S. aureus* without promoting antibiotic resistance.

## Materials and methods

### Ethics statement

The study adhered to ARRIVE guidelines (https://arriveguidelines.org), and all protocols for antibody production were approved by the ethical board of the University of Pavia. Protocols for the *in vivo* experiments were conducted in accordance with University of Messina institutional and national ethical guidelines.

### Bacterial strains and culture conditions

All strains used in this study are listed in Table 1. *S. aureus* cells were grown to stationary phase in brain heart infusion (BHI) (VWR International Srl, Milan, Italy) at 37 °C with shaking. Vector pFnBA4 was transferred to *S. aureus* SH10004X that were made electrocompetent as previously described [32,52] grown in BHI agar and BHI broth containing 10 μg/ml chloramphenicol (Merck, Darmstadt, Germany). *Escherichia coli* BL21 (DE3) (Invitrogen, Carlsbad, CA, USA), TOP10 (Thermo Scientific, MA, USA) and IMO8B [53] cells were grown in Luria agar and Luria broth (VWR International, PA, USA) containing 100 μg/ml ampicillin (Merck) at 37 °C with shaking. BL21 cells were transformed with vector pQE30 (Qiagen Aarhus, Denmark), while TOP10 and IMO8B were transformed with vector pFnBA4 derivatives. In those experiments, where a defined number of cells were used, bacteria were harvested from the cultures by centrifugation, washed, resuspended in phosphate-buffered saline (PBS) at the suitable density by spectrophotometer measurements at A_600nm_ (Jasco, V-630).

**Table 1:** Bacterial strains used in this study.

Bacterial strain	Relevant properties	Reference
*S. aureus*		
SH1000 4X	SH1000 is a *S. aureus* strain 8325–4 derivative with a restored *rsbU* gene. SH1000 4X is a quadruple mutant deficient in *clfA, clfB, fnbA* and *fnbB*.	[7,31]
SH10004X (pFnBA4_WT)	SH1000 4X derivative carrying pFnBA4 overexpressing wild-type *fnbA;* ampicillin and chloramphenicol resistance	[31,54]
SH10004X (pFnBA4_Q103A)	SH10004X (pFnBA4) derivative encoding DNA for a Q103A substitution within FnBPA (CmR)	This study
*E. coli*		
BL21 (DE3)	Cloning host	Invitrogen
XL1Blue	Cloning host	Agilent
TOP10	Cloning host	Thermo Scientific
IMO8B	Plasmid propagation for transformation into *S. aureus* (SA08BΩPN25-*hsdS*)	[53]

### Plasmid and DNA manipulation

#### Production of constructs for recombinant protein expression.

The DNA fragment encoding FnBPA N1 Q103A (residues 37–193) was purchased from Eurofins Genomics. This DNA fragment (insert) was amplified with the following forward (5’-TCACGGATCCGCATCAGAACAAAAGACAAC-3’) and reverse (5’-ACGTCGACCTACGTTTCCACTTTCGCGTTAC-3’) primers, digested alongside the vector (pQE30) with BamHI and SalI restriction enzymes and ligated with T4 DNA ligase (Thermo Scientific) to obtain the pQE30-N1-Q103A plasmid.

The N1N2N3 Q103A mutation was obtained through a fusion PCR in two steps: firstly, the N1 Q103A fragment was obtained using pQE30-N1-Q103A template, with forward (5’-TCACGGATCCGCATCAGAACAAAAGACAAC-3’) and reverse (5’-GTTACATCTGTACCCGTTTCCACTTTCGCGTTAC-3’) primers and the N2N3 fragment was obtained using pFnBPAN1–3 template obtained in our previous work [27] with forward (5’-GTAACGCGAAAGTGGAAACGGGTACAGATGTAAC-3’) and reverse (5’-CTGCAGGTCGACCTAATTTTTCTCATTTCCGTTC-3’) primers. The two fragments were then used as templates to obtain the N1N2N3 Q103A mutant, using forward (5’-TCACGGATCCGCATCAGAACAAAAGACAAC-3’) and reverse (5’-CTGCAGGtCGACCTAATTTTTCTCATTTCCGTTC-3’) primers, thus generating the insert. The insert and the vector (pQE30) were digested and ligated as reported above.

All constructs were verified by sequencing using primers pQEfor (5’-GTATCACGAGGCCCTTTCGTCT-3’) and pQErev (5’- CATTACTGGATCTATCAACAGGAG-3’).

#### Site-directed mutagenesis for mutant pFnBA4 production.

Plasmid pFnBA4 Q103A was constructed using overlapping primers in two rounds of PCR amplification. The first round used forward primer (5′-CTCAAGACAATAGCGGAGA**T**GAAAGACAAGTAGATTTAAC-3′), and reverse primer (5′-GTTAAATCTACTTGTCTTT**C**ATCTCCGCTATTGTCTTGAG-3′), Phusion (0.5 U, NEB) and Phirehotstart II (Thermo Scientific 0.25 U) DNA polymerases were used for amplification as previously described [55] with denaturation 98°C for 2 min, 18 cycles of 98°C for 30 s, 55°C for 30 s, and 72°C for 15 s per kb with a final extension of 72°C, 10 min.

The amplification product was treated with DpnI enzyme (NEB) for 1 h at 37˚C and used to transform TOP10 cells. Plasmid was extracted from TOP10 and sequenced. Plasmid carrying the correct substitution was used as template for a second round of site-directed mutagenesis using the procedure described above and primers (5′-CAATCTCAAGACAATAGCGGAGAT**A**AAAGACAAGTAGATTTAACAC-3′) (forward) and (5′-GTGTTAAATCTACTTGTCTTT**T**ATCTCCGCTATTGTCTTGAGATTG-3′) (reverse). Amplification products were treated in the manner described above and used to transform TOP10. The Q103A substitution was confirmed by sequencing plasmid isolated from TOP10 (PlasmidsNG).

### Multiple sequence analysis

Sequences encoding FnBPA and FnBPB from several *S. aureus* clonal complexes were obtained from KEGG Genomes. Sequence alignment of the DNA sequences was performed in ClustalOmega Multiple Sequence Alignment (MSA) tool.

### Expression and purification of recombinant proteins

Recombinant mutant FnBPA N1N2N3 Q103A and N1 Q103A regions were expressed using the pQE30 plasmid in *E. coli* BL21 (DE3). An overnight starter culture was diluted 1:40 in Luria broth containing ampicillin and incubated with shaking until reaching the exponential growth phase (OD_600nm_ = 0.4–0.6). Protein expression was induced by adding isopropyl 1-thio-β-D-galactopyranoside (IPTG) (Merck) to a final concentration of 1 mM, followed by overnight incubation at 28 °C. Bacterial cells were harvested by centrifugation and stored at −80 °C. For protein extraction, the cells were resuspended in a lysis buffer (50 mM NaH_2_PO_4_, 300 mM NaCl, pH 8.0, supplemented with 1 mM MgCl2, 1 mM phenylmethylsulfonyl fluoride (PMSF, Merck), and 20 μg/mL protease-free DNase I (Merck) and lysed by sonication (70% amplitude, 12 cycles of 30 seconds on/off with 1 minute 30 seconds intervals). Cell debris was removed by centrifugation, and the recombinant proteins were purified from the supernatant using Ni² ⁺ -affinity chromatography on a HiTrap chelating column (GE Healthcare, Buckinghamshire, UK). Protein purity was confirmed by SDS-PAGE followed by Bio-Safe Coomassie staining (BioRad, Hercules, CA, USA). The concentration of the purified proteins was determined using a bicinchoninic acid (BCA) protein assay (Pierce, Rockford, IL, USA).

The wild type N1N2N3, N1 FnBPA regions and vWbp were already produced in our previous work with the same protocol as described above.

### Reagents, proteins and antibodies

Bovine serum albumin (BSA) and skim milk were obtained from Merck. Human prothrombin, human factor XIII (FXIII), and fibrinogen (Fbg) were sourced from Prolytix (VT, USA), while transglutaminase-2 (TG2) was purchased from Zedira GmbH (Darmstadt, Germany). Polyclonal antibodies against FnPBA, and *S. aureus* were raised in mice using purified bacterial proteins as antigens, following standard immunization procedures. Polyclonal anti-Fbg antibody was also produced following standard immunization procedures. The study adhered to ARRIVE guidelines (https://arriveguidelines.org), and all protocols for antibody production were approved by the ethical board of the University of Pavia. The secondary rabbit anti-mouse horseradish peroxidase (HRP)-conjugated antibody was sourced from Dako Cytomation (Glostrup, Denmark), and o-phenylenediamine dihydrochloride (OPD) tablets were obtained from Thermo Scientific. The HRP conjugated rabbit anti *S. aureus* alpha-toxin polyclonal antibody was purchased from EMELCA Bioscience (Netherlands).

### Western blot assays

#### Cross‑linking of recombinant FnBPA proteins to Fbg promoted by vWbp‑activated FXIII.

To assess the cross-linking of recombinant wild type and mutant FnBPA regions (N1N2N3, N1N2N3 Q103A, N1 and N1 Q103A) to fibrin(ogen) mediated by vWbp-activated FXIII, Western immunoblotting assays were conducted. Reaction mixtures containing 200 nM prothrombin (ProT), 200 nMvWbp, 15 µg/mL FXIII, 5 µM Fbg, and 2.5 µM of each bacterial protein were incubated at 37 °C in 25 mM Tris-HCl buffer (pH 7.5) with 5 mM CaCl₂ for specified time intervals. The reactions were terminated by adding a stop buffer containing 62.5 mM Tris-HCl (pH 6.8), 4 M urea, 2% SDS, 10% glycerol, 5% β-mercaptoethanol, and 0.01% bromophenol blue. Samples were heated at 98 °C for 5 minutes, separated using SDS-PAGE on 4.8–10% polyacrylamide gels, and transferred onto PVDF membranes. Following overnight blocking with 5% (w/v) skim milk and washing, the membranes were immunostained with specific mouse antibodies against FnBPA. HRP-conjugated rabbit anti-mouse IgG (1:10 000 dilution) was used as the secondary antibody, and the blots were developed using the Westar Supernova detection kit (Cyanagen srl, Bologna, Italy). An ImageQuant LAS 4000 mini-biomolecular imager (GE Healthcare) was used to capture the images.

#### Cross‑linking of recombinant FnBPA proteins to Fbg promoted by TG2.

To examine the covalent bond formation between FnBPA and Fbg catalyzed by TG2, 2.5 µM of each recombinant FnBPA wild type or mutant region region (N1N2N3, N1N2N3 Q103A, N1 and N1 Q103A) was incubated with 5 µM Fbg in the presence of 50 nM TG2. The reactions were carried out in 25 mM Tris-HCl buffer (pH 7.5) containing 10 mM CaCl₂ for specified durations at 37°C. Samples were then heated at 98 °C for 5 minutes, resolved by SDS-PAGE on 4.8–10% polyacrylamide gels, and transferred onto PVDF membranes. After overnight blocking with 5% (w/v) skim milk, the membranes were washed and immunostained with a mouse polyclonal antibody specific to FnBPA, diluted 1:5000. The bound antibodies were detected using an HRP-conjugated rabbit anti-mouse IgG (1:10,000) and the blotted membrane was developed as described above.

#### Production of alpha-toxin by *S. aureus* SH1000 4X (pFnBA4_WT) or SH1000 4X (pFnBA4_Q103A).

To evaluate the secretion levels of Hla in *S. aureus* culture medium, bacterial cells were cultured in BHI medium. Following centrifugation, supernatants were filtered (pore size 0,22 μm) and concentrated at 4°C approximately 10X through Amicon Ultra centrifugal devices (MWCO 10 kDa). Five μl of each sample were loaded on a 12.5% SDS-PAGE gel and transferred onto a PVDF membrane (BioRad). After overnight incubation at 4°C with 5% skim milk (w/v) in PBST, the membrane was treated for 45’ at 22°C with 0.5 µg/mL HRP conjugated rabbit anti-Hla antibody in 1% skim milk (w/v). Following several washings with PBST, blots were developed using the Westar Supernova detection kit (Cyanagen srl, Bologna, Italy). An ImageQuant LAS 4000 mini-biomolecular imager (GE Healthcare) was used to capture images of the bands. The signal intensities were quantified with ImageJ and plotted on GraphPad Prism.

### ELISA assays

#### Saturation binding curves of FnBPA N1N2N3 wild type or Q103A to fibrinogen.

To test if the binding affinity of the wild type and mutant form of the region A of FnBPA is maintained, we constructed saturation binding curves. Microtiter wells were coated with 1µg/well of each of the recombinant protein and immobilized overnight at 4°C. The wells were washed three times with 0.5% (v/v) Tween-20 in PBS (PBST) and treated for 1 h at room temperature with 2% (v/v) BSA in PBS. After that, 1:2 serial dilutions of Fbg in PBS (starting from 4µM) were added to the wells and incubated again for 1h at room temperature. The binding of Fbg to FnBPA was assessed by incubating the wells with a mouse polyclonal anti-Fbg IgG (1:1000) diluted in 1% (v/v) BSA for 1 h. This was followed by a 45 min incubation with an HRP-conjugated anti-mouse IgG (1:1000), also diluted in 1% (v/v) BSA. After thorough washing, OPD was added to the wells, and the absorbance at 490 nm was measured using an ELISA plate reader (Steroglass).

#### Cross‑linking quantification of recombinant FnBPA proteins to Fbg promoted by vWbp‑activated FXIII.

To confirm and quantify the Western immublotting results, the crosslinking of different FnBPA wild type or mutant regions (N1N2N3, N1N2N3 Q103A, N1 and N1 Q103A) was performed through and ELISA assay. Microtiter wells were coated with 3µg/ml of Fbg in PBS and incubated overnight at 4°C. After the 2% (v/v) BSA treament, the wells were coated with mixtures prepared in 25 mM Tris-HCl buffer (TBS) (pH 7.5), containing 200 nM prothrombin (ProT), 200 nM vWbp, 15 µg/mL FXIII, and 300nm of each FnBPA form, added with either 2 mM CaCl₂ or 2 mM EDTA. The wells were then incubated at 37°C for 1 h, and the binding was revealed with a mouse polyclonal anti-FnBPA IgG (1:1000) diluted in 1% (v/v) BSA for 1 hour, followed by a 45-minute incubation with an HRP-conjugated anti-mouse IgG (1:1000), also diluted in 1% (v/v). The absorbance values were obtained as reported above.

#### Cross‑linking quantification of recombinant FnBPA proteins to Fbg promoted by TG2.

To validate and quantify the Western blotting results, the crosslinking of various FnBPA regions (wild-type and mutants), including N1N2N3, N1N2N3 Q103A, N1, and N1 Q103A, was assessed using an ELISA assay. Microtiter wells were coated with 3 µg/mL Fbg in PBS and incubated overnight at 4 °C. Following 2% (v/v) BSA treatment, the wells were treated with mixtures prepared in 25 mM Tris-HCl buffer (pH 7.5) containing 50 nM TG2 and 300 nM of each FnBPA variant, supplemented with either 10 mM CaCl₂ or 2 mM EDTA. The plates were incubated at 37 °C for 1 hour, after which binding was detected using a mouse polyclonal anti-FnBPA IgG (1:1000) diluted in 1% (v/v) BSA for 1 hour, followed by a 45-minute incubation with an HRP-conjugated anti-mouse IgG (1:1000), also diluted in 1% (v/v) BSA. Absorbance values were measured as described previously.

#### Binding of *S. aureus* SH1000 4X (pFnBA4) wild type or Q103A to Fbg.

To test if the mutant *S. aureus* strain retains the same binding interaction to Fbg as the wild type strain, another ELISA assay was performed. Microtiter wells were coated with 1:2 serial dilution of Fbg in PBS, starting from 1 µg/well, and incubated overnight at 4 °C. Simultaneously, *S. aureus* SH10004X::pFnBA4 wild-type or mutant strains were cultured to stationary phase and diluted the following day to an OD₆₀₀ of 1. After blocking with 2% (v/v) BSA, the wells were treated with 100 µL of each diluted bacterial solution and incubated at 37 °C for 1 hour. Binding was detected using a mouse polyclonal anti-*S. aureus* IgG (1:1000) diluted in 1% (v/v) BSA for 1 hour. This was followed by a 45-minute incubation with an HRP-conjugated anti-mouse IgG (1:1000), also diluted in 1% (v/v) BSA. Absorbance values were measured as described previously.

#### Cross‑linking of *S. aureus* SH1000 4X (pFnBA4) wild type or Q103A to Fbg promoted by vWbp-activated FXIII.

To test whether the Q103A mutation can inhibit the bacterial incorporation into the fibrin mesh through the covalent interaction between Fbg and FnBPA, another ELISA assay was performed. Microtiter wells were coated with 3 µg/mL Fbg in PBS and incubated overnight at 4 °C. Simultaneously, *S. aureus* SH1000 4X (pFnBA4) wild type or mutant strains were grown to stationary phase and the next day diluted to an OD_600nnm_ = 1. Following 2% (v/v) BSA treatment, the wells were treated with mixtures prepared in 25 mM Tris-HCl buffer (TBS, pH 7.5) containing 200 nM prothrombin (ProT), 200 nM vWbp, 15 µg/mL FXIII, and 100 µL of each diluted bacterial solution, supplemented with either 2 mM CaCl₂ or 2 mM EDTA. The wells were incubated at 37 °C for 1 hour, and binding was detected using a mouse polyclonal anti-*S. aureus* IgG (1:1000) diluted in 1% (v/v) BSA for 1 hour. Subsequently, the wells were incubated for 45 minutes with an HRP-conjugated anti-mouse IgG (1:1000) prepared in 1% (v/v) BSA. The absorbance values were then measured as outlined above.

### Mouse model of *S. aureus*-induced dermonecrosis

To assess the *in vivo* impact of the Q103A mutation in FnBPA on *S. aureus* pathogenicity, a murine dermonecrosis model was employed [33]. Female C57BL/6 mice (6–8 weeks old) were injected intradermally with 10⁷ CFU of *S. aureus* SH10004X strains expressing either wild-type or Q103A-mutated FnBPA. Infections were carried out under anaesthesia induced by intraperitoneal injection of tiletamine/zolazepam (40 mg/kg) and xylazine (10 mg/kg), in accordance with institutional and national ethical guidelines.

At day 7 post-infection, lesion areas were measured, and skin biopsies were collected from the site of infection for CFU quantification. Bacterial load was determined by homogenizing tissue in sterile PBS, followed by serial dilution and plating on TSA plates.

Lesion size (mm²) and CFU/biopsy data were analyzed using the Mann–Whitney U test. Results showed that mice infected with the wild-type strain developed significantly larger necrotic lesions and higher bacterial burden compared to those infected with the Q103A mutant (*p* < 0.01). All animal studies were performed in strict accordance with the European Union guidelines for the use of laboratory animals. The procedures were approved by the Animal Welfare Committee (OPBA permit no. 18052010) of the University of Messina and by the Ministero della Salute of Italy (permits no. 665/2015 and no. 786/2018-PR prot. 5E567.10).

### Statistical methods

Data analyses were performed using Prism 4.0 (GraphPad). Each experiment was conducted with at least three biological replicates (three independent experiments). To compare two groups, a two-tailed Student’s t-test was applied. P values less than 0.05 were considered statistically significant, with the following symbols used to denote significance: *P < 0.05, **P < 0.01, and ***P < 0.001.

## Supporting information

S1 Fig Conservation and functional relevance of the Q103 residue in FnBPA isoforms. Multiple sequence alignment was conducted for fnbA genes from S. aureus strains (reported on the left) harbouring the 7 isoforms of FnBPA [30] to determine the conservation of the Q103 residue (in red), implicated in bacterially-mediated cross-linking with fibrinogen. Alignments were carried out with ClustalOmega.
(TIF)

S2 Fig Production of bacterial αlpha-toxin (Hla) assayed by western blot. *S. aureus* SH10004X (pFnBPA4_WT) or SH10004X (pFnBPA4_Q103A) cells were cultured in BHI medium. Bacterial supernatants were loaded onto 12.5% SDS-PAGE and transferred onto a PVDF membrane. Protein levels were detected through rabbit HRP-conjugated anti-Hla antibody (0.5 µg/ml in milk 1%). Values are results of densitometric analysis obtained through ImageJ and represented as percent of control (SH10004X (pFnBPA4_WT), set at 100%. Represented data are representative of three independent experiments. Data were analyzed through t-test statistical analysis.
(TIF)

S1 File Summary diagram of this study. (PDF)

S2 File Raw data for all the results of this study. (XLSX)
