# Supplementary figures and images for: A single amino acid substitution in Fibronectin Binding protein A (FnBPA) governs Staphylococcus aureus virulence via host transglutaminase-mediated fibrin crosslinking
Source: PLoS Pathog. 2025 Dec 1;21(12):e1013743. doi: 10.1371/journal.ppat.1013743 (PMC12680339; doi:10.1371/journal.ppat.1013743)

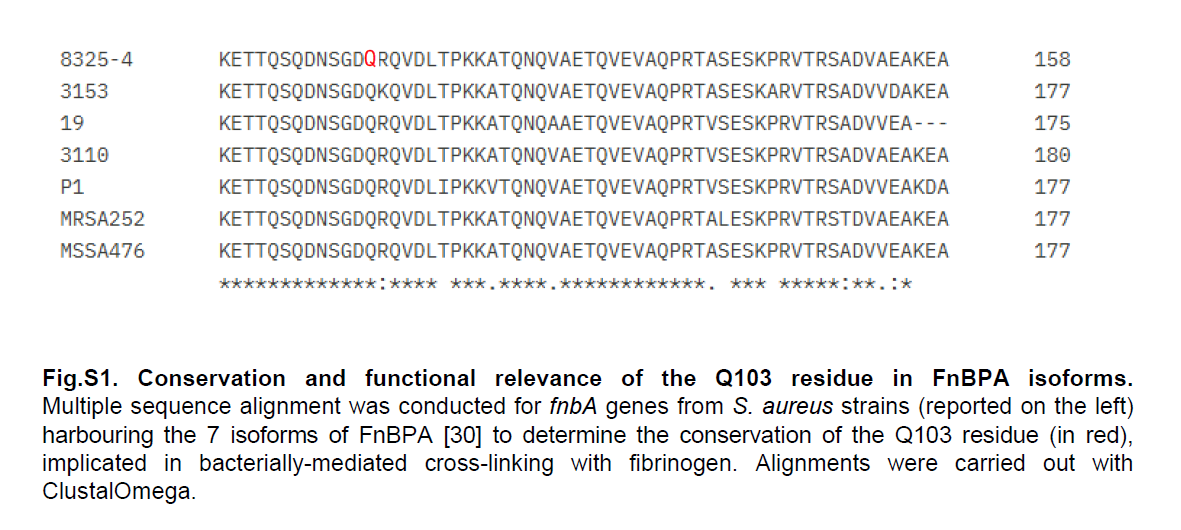

Supplement: S1 Fig — Multiple sequence alignment was conducted for fnbA genes from S. aureus strains (reported on the left) harbouring the 7 isoforms of FnBPA [30] to determine the conservation of the Q103 residue (in red), implicated in bacterially-mediated cross-linking with fibrinogen. Alignments were carried out with ClustalOmega. (TIF) [file ppat.1013743.s001.tif]

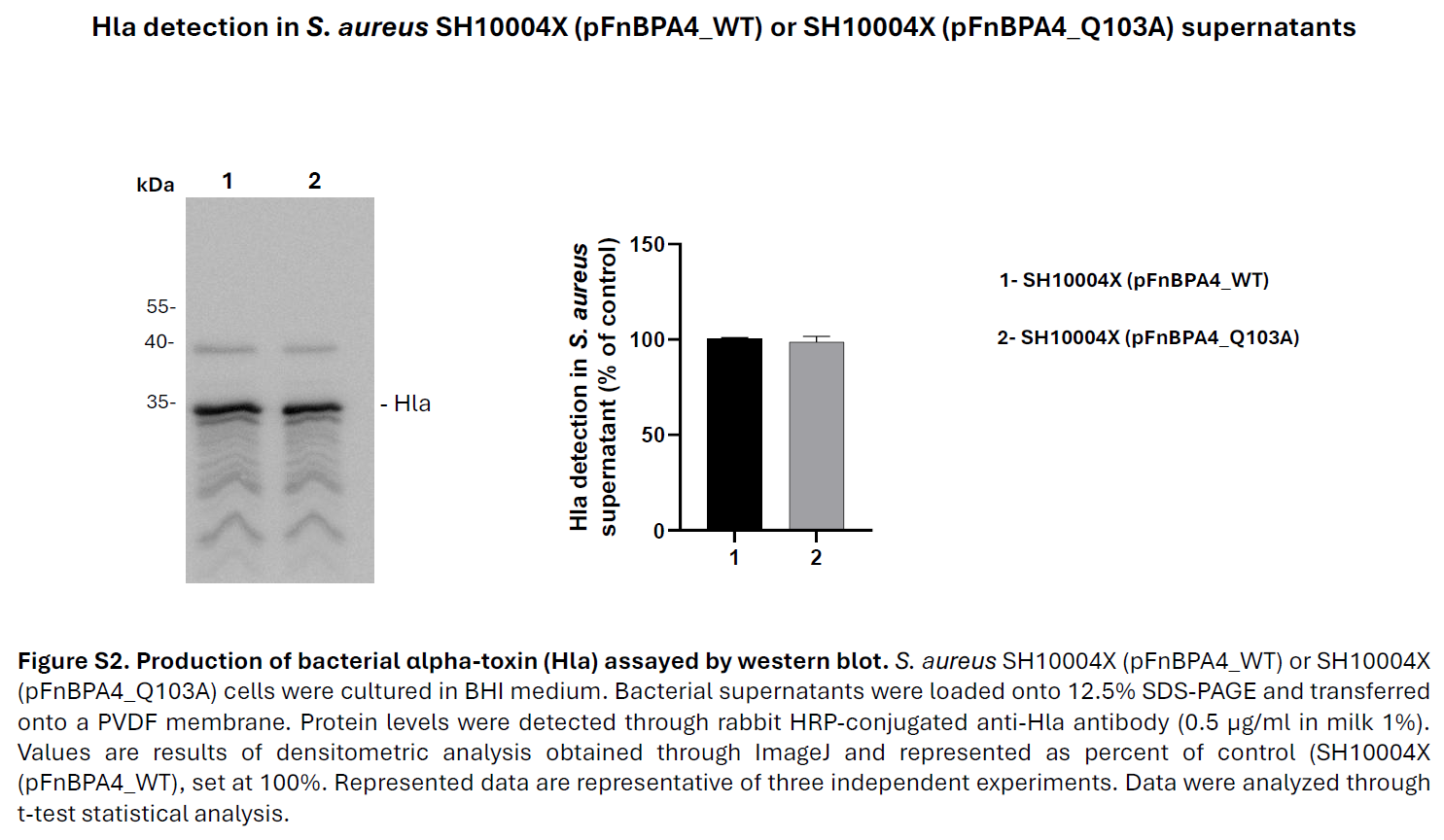

Supplement: S2 Fig — S. aureus SH10004X (pFnBPA4_WT) or SH10004X (pFnBPA4_Q103A) cells were cultured in BHI medium. Bacterial supernatants were loaded onto 12.5% SDS-PAGE and transferred onto a PVDF membrane. Protein levels were detected through rabbit HRP-conjugated anti-Hla antibody (0.5 µg/ml in milk 1%). Values are results of densitometric analysis obtained through ImageJ and represented as percent of control (SH10004X (pFnBPA4_WT), set at 100%. Represented data are representative of three independent experiments. Data were analyzed through t-test statistical analysis. (TIF) [file ppat.1013743.s002.tif]
