# Supplementary material for: A single amino acid substitution in Fibronectin Binding protein A (FnBPA) governs Staphylococcus aureus virulence via host transglutaminase-mediated fibrin crosslinking
Source: PLoS Pathog. 2025 Dec 1;21(12):e1013743. doi: 10.1371/journal.ppat.1013743 (PMC12680339; doi:10.1371/journal.ppat.1013743)
Supplement: S1 File — (PDF) [file ppat.1013743.s003.pdf]

# Summary Diagram

## 1. Initial binding mechanism

*S. aureus* expresses **FnBPA** (fibronectin-binding protein A)

↓

FnBPA binds non-covalently to **fibrinogen** via "Dock, Lock, and Latch" (DLL) mechanism

↓

This interaction is essential for colonization of host tissues

## 2. Covalent crosslinking of FnBPA to fibrin(ogen)

- **Pathway A: Clotting-related**

**vWbp** (von Willebrand factor binding protein) from *S. aureus*

↓

Activates host **Factor XIII (FXIII)** → **FXIIIa**

↓

Crosslinks **FnBPA** (through the Q103 residue) to fibrin(ogen)

- **Pathway B: Tissue-associated**

Host **TG2** (Tissue Transglutaminase 2)

↓

Crosslinks **FnBPA** to fibrin(ogen)

## 3. Key residue Q103 in FnBPA

- Located in **N1 subdomain** of FnBPA
- Required for covalent crosslinking
- Mutation of **Q103A**:

✓ **Preserves** non-covalent binding

✖ **Prevents** covalent crosslinking

## 4. Experimental Evidence

| Experiment | Observation |
|---|---|
| Recombinant protein (wild-type or Q103A) binding to fibrinogen | Normal non-covalent binding to Fbg |
| Western blot & ELISA | Q103A mutant fails to form high molecular weight complexes |
| Bacterial surface expression | *S. aureus* expressing Q103A cannot be covalently incorporated into fibrin |
| Mouse infection model | *S. aureus* expressing Q103A causes smaller lesions and lower bacterial loads than strain expressing wild-type FnBPA |

## 5. Virulence Impact and therapeutic implications

**- WT FnBPA**: Efficient fibrin incorporation → strong tissue colonization → high virulence

**- Q103A mutant FnBPA**: Impaired fibrin anchoring → reduced colonization → attenuated virulence

↓

Targeting **FnBPA Q103 crosslinking of fibrin(ogen)** offers:

- A potential anti-virulence strategy
- A novel target for treating antibiotic-resistant *S. aureus* infections

## 6. Overall Conclusion

*S. aureus* exploits host transglutaminases (FXIIIa & TG2) to covalently anchor itself to fibrin(ogen) via FnBPA Q103 residue, enhancing its ability to adhere and cause tissue damage. Blocking this interaction reduces virulence and offers a promising therapeutic target.
